# Future-relevant memories are not selectively strengthened during sleep

**DOI:** 10.1371/journal.pone.0258110

**Published:** 2021-11-04

**Authors:** Jennifer E. Ashton, Scott A. Cairney

**Affiliations:** 1 Department of Psychology, University of York, York, United Kingdom; 2 York Biomedical Research Institute, University of York, York, United Kingdom; University of Utah, UNITED STATES

## Abstract

Overnight consolidation processes are thought to operate in a selective manner, such that important (i.e. future-relevant) memories are strengthened ahead of irrelevant information. Using an online protocol, we sought to replicate the seminal finding that the memory benefits of sleep are enhanced when people expect a future test [Wilhelm et al., 2011]. Participants memorised verbal paired associates to a criterion of 60 percent (Experiment 1) or 40 percent correct (Experiment 2) before a 12-hour delay containing overnight sleep (sleep group) or daytime wakefulness (wake group). Critically, half of the participants were informed that they would be tested again the following day, whereas the other half were told that they would carry out a different set of tasks. We observed a robust memory benefit of overnight consolidation, with the sleep group outperforming the wake group in both experiments. However, knowledge of an upcoming test had no impact on sleep-associated consolidation in either experiment, suggesting that overnight memory processes were not enhanced for future-relevant information. These findings, together with other failed replication attempts, show that sleep does not provide selective support to memories that are deemed relevant for the future.

## Introduction

It is now well established that sleep supports the consolidation of newly acquired declarative memories [1–4]. Sleep consistently results in better memory retention when compared to equivalent periods of wakefulness [1,2,5–10] and these behavioural effects have been associated with specific features of sleep (e.g. slow-wave sleep [11–13]). Contemporary models of sleep-associated consolidation suggest that newly formed memories are reactivated during sleep, prompting their migration from hippocampus to neocortex for long-term storage [1,2,13,14]. However, new memories do not all profit equally from sleep (i.e. some memories benefit more from sleep than others), and the factors that determine to what extent a memory will benefit from overnight consolidation are not fully understood.

There is growing evidence to suggest that sleep offers special protection to salient information. For example, the benefits of sleep are enhanced for memories that are considered emotionally negative [15–19], that are associated with monetary reward [20] or that are deemed relevant for the future [21–23]. The perceived relevance of newly learned information can be manipulated experimentally by controlling test expectancy; that is, whether or not an individual expects their memory to be assessed. In their seminal study, Wilhelm et al. [24] trained participants on a declarative memory task before a delay containing sleep or wakefulness. Crucially, half of the participants were informed that they would be re-tested in a later session, whereas the other half were told that they would complete a different set of tasks. Among participants that slept (but not those who remained awake), declarative memory retention was better for individuals who expected a test, as compared to those who did not, suggesting that sleep had selectively strengthened future-relevant information.

However, other findings concerning the selective influences of sleep have been somewhat contradictory. A number of studies have failed to observe a preferential benefit of sleep on emotionally negative relative to neutral memories [6,25–30], and others have observed no impact of monetary reward on sleep-associated consolidation [31–33]. In recent work, knowledge of a future test had no impact on the overnight retention of declarative or nondeclarative memories [34], and failed to influence the neural correlates of memory retention after sleep [35]. The circumstances by which future expectations affect the salience of newly acquired information, and the subsequent impacts on sleep-associated memory processing, therefore remain unclear.

To address this issue, we sought to replicate the seminal findings of Wilhelm et al. [24] in an online protocol and test the hypothesis that the benefits of sleep for memory are amplified when newly learned information is deemed relevant for the future. In Experiment 1, participants completed verbal paired associates training (encoding and baseline test) before a 12-hour retention interval containing overnight sleep or daytime wakefulness. Critically, after training, half of the participants were informed that they would be tested again in the following session, whereas the other half were told to expect a different set of tasks. All participants were re-tested 12 hours later, and again after 7 days, allowing us to study the impact of test expectancy on sleep-associated consolidation and to determine the extent to which these effects are maintained in the longer term. Because participants received feedback on their final round of baseline testing, we expected to observe an improvement in memory performance between the baseline and follow-up tests. Importantly, however, we also expected that the extent of this improvement would be greater after sleep than wakefulness and, furthermore, that this sleep-associated memory gain would be augmented for individuals who had knowledge of a future test.

There is growing evidence to suggest that sleep favours the consolidation of weakly-encoded relative to strongly-encoded memories [36–38]. We therefore reasoned that if baseline performance levels are too high, then knowledge of a future test might not augment sleep-associated consolidation. We tested this possibility in our second, pre-registered experiment (osf.io/3k6ej), by adjusting our paired associates memory paradigm to reduce pre-sleep performance.

## Experiment 1

### Methods

#### Participants

Two-hundred and thirty adults (163 women, mean ± SD age: 24.89 ± 3.66 years) were recruited online via Prolific (https://app.prolific.co/) and reported to be living in the UK with English as their first language. On the days of the experiment, participants were asked to abstain from alcohol and avoid taking naps. Informed consent was obtained from all participants in line with the Research Ethics Committee of the Department of Psychology at the University of York. Only participants who expected/did not expect a memory test in line with their respective condition were included in the final analysis (e.g. individuals in the unexpected condition who reported to have expected a follow-up test after the baseline test were excluded; see *Follow-up questionnaires* below). The number of participants excluded from each condition was: sleep-expected *n* = 12, sleep-unexpected *n* = 19, wake-expected *n* = 10, wake-unexpected *n* = 25. A further two participants were excluded; one for reporting to have used pen and paper to memorise the stimuli, and another who reported napping during the day of the study. This resulted in a final sample of 162 participants (117 women, mean ± SD age: 25.04 ± 3.74 years) who were allocated to one of four conditions: sleep-expected (*n* = 42), sleep-unexpected (*n* = 39), wake-expected (*n* = 50) or wake-unexpected (*n* = 31). Assignment to the expected and unexpected conditions was determined by the time that participants signed up for the study (AM or PM), which alternated between testing days. Evening sign ups were always allocated to the sleep group and morning sign ups were always allocated to the wake group.

Our sample size was calculated using an effect size reported in Wilhelm et al. [24]. The effect of interest (*d* = 0.86) was derived from a t-test comparing memory retention after sleep in individuals who did and did not expect a test. We determined that a minimum sample size of *n* = 120 (*n* = 30 per condition) would be necessary for 95% power (one-tailed, alpha = .05) to detect an effect of this magnitude. Data collection continued until our desired sample size was met, with each participant providing a full and usable data set.

#### Procedure

**Fig 1:**
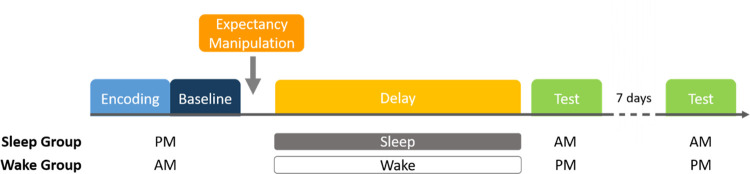
Study procedure. All participants completed a verbal paired associates task (encoding and baseline test) in the morning (wake groups) or evening (sleep groups). Afterwards, participants were informed that their memory for the word pairs would be tested in the next session (expected condition) or were told to expect a different set of tasks (unexpected condition). All participants were tested again 12 hours and 7 days after encoding.

A schematic of the study is shown in Fig 1. All participants completed verbal paired associates training (encoding and baseline memory test) in the morning (between 7am and 9am; wake groups) or evening (between 7pm and 9pm; sleep groups). Participants then returned 12 hours and 7 days after encoding to complete follow-up tests.

Critically, after the baseline test, half of the participants (in both the wake and sleep groups) were informed that their memory for the pairs would be tested in the next session (expected condition), whereas the other half were told they would carry out a different set of tasks (unexpected condition).

*Encoding and baseline assessment*. The paired associates task required participants to learn 40 semantically-related word pairs (e.g. *horizon*–*sun*). On each encoding trial, a randomly selected word pair was presented in the centre of the screen for 5 s, followed by an inter-stimulus interval of 200 ms. Participants were instructed to commit each pair to memory by imagining a scenario in which the words were interacting (for example, one might imagine the sun coming up over the horizon in the example above). Baseline memory performance was assessed immediately after encoding using a cued-recall procedure (for all 40 word pairs). On each trial, the first word (cue) of a randomly selected pair was presented on the centre left of the screen and participants were required to type the associated second word (target). Participants had 10 s to provide each response and submitted their answer by pressing ‘enter’ on the keyboard. Following each response, and irrespective of accuracy, the cue and target words were displayed together for 2 s. Participants were instructed to use this feedback to relearn any pairs that they may have recalled incorrectly. This cued-recall procedure was repeated for a second time if participants had not reached a criterion of 60% correct responses in the first baseline test (all 40 word pairs were re-tested). Participants completed the test a maximum of two times (55% of participants completed a second test). The proportion of participants who completed a second test did not differ between the sleep and wake groups, *X*^*2*^ (1, *N* = 162) = 0.62, *p* = .430.

*Expectancy manipulation and retention interval*. Following the baseline memory assessment, participants in the ‘expected’ groups were told that they would be retested in the next session, whereas participants in the ‘unexpected’ groups were told they would perform a different set of tasks. To avoid active rehearsal of the word pairs, all participants completed a puzzle matching game for 5 minutes before finishing the session. Participants then entered a 12-hour retention interval, which took place across the day (wake groups) or night (sleep groups). Participants in the wake groups followed their usual daytime routine and abstained from taking naps. Participants in the sleep groups followed their usual home sleep routine. Participants were not given any explicit instructions about the tasks that they would perform at the final follow-up (7 days later).

*Memory retrieval*. Participants returned 12 hours and 7 days after the first session. During these follow-up sessions, their memory for all 40 word pairs was retested using the same cued-recall procedures as at the baseline session, with the exception that no feedback was provided.

*Follow-up questionnaires*. Participants completed questionnaires probing the amount of sleep that they had achieved during the 12-hour delay (sleep groups), or whether they had taken any naps (wake groups). Critically, all participants reported whether or not they had expected to be retested on the word pairs after the baseline test. Participants in the unexpected conditions who reported to have expected a test were excluded, as were participants in the expected conditions who reported to have not expected a test. As outlined above (see *Participants*), the majority of individuals excluded on this basis were those in the unexpected condition who reported to have expected another test after the baseline test. A total of 66 participants were excluded on this basis in Experiment 1 (see *Participants*).

*Alertness*. Alertness was assessed at the beginning of each session using the Stanford Sleepiness Scale (SSS [39]) and a Psychomotor Vigilance Task (PVT). During the PVT, participants were presented with a blank grey screen. At random intervals, a red cross appeared in the centre of the screen and participants were required to press the keyboard space bar as quickly as possible. Inter-stimulus intervals were randomly distributed from 2 to 10 s and the task lasted for a total of 3 minutes.

#### Analysis

Memory retention at each follow-up test was defined as the percentage of correctly recalled word pairs, with performance on the final round of the baseline test set to 100% [24]. For example, if a participant recalled 30 out of 40 words at baseline and then 35 out of 40 words at the 12-h follow-up, then their 12-h retention score would be ~117% ([35/30]*100). Owing to the feedback provided during the final baseline assessment, cued-recall performance at the follow-up tests was expected to be above 100%. Retention scores at the 12-hour and 7-day tests were applied to separate 2 (Delay: Sleep/Wake) * 2 (Expectancy: Expected/Unexpected) ANOVAs. To assess the strength of non-significant effects, a corresponding factorial Bayesian ANOVA was calculated for each test with the same factors as above. By convention, the strength of evidence for the null hypothesis, in comparison to the experimental hypothesis, is regarded as noteworthy if the Bayes Factor (BF_01_) is > 3 [40].

Alertness in each session was measured using scores provided on the SSS [39]. Vigilance was assessed by the number of attentional lapses (i.e. trials with a response time [RT] > 500 ms) made during the PVT [41]. These measures were subjected to separate 2 (Delay: Sleep/Wake) * 2 (Expectancy: Expected/Unexpected) ANOVAs at each session. All analyses were completed in *R* [42] using the *Rstatix* [43] package.

## Results

### Baseline memory performance

Across all conditions, baseline memory performance (calculated as the percentage of correctly recalled word pairs in the final baseline test) was 77% ± 0.95 (mean ± SEM; see Table 3). A 2 (Delay: Sleep/Wake) * 2 (Expectancy: Expected/Unexpected) ANOVA showed no group differences at baseline (Delay: *F*(1,158) = 0.10, *p* = .320, Expectancy: *F*(1, 158) = 0.12, *p* = .726) and no Delay*Expectancy interaction (*F*(1, 158) = 0.02, *p* = .900). Nine participants failed to reach the performance criterion of 60% after two attempts. The lowest score among these participants was 37.5% and the average score was 48%. Our results did not change when we repeated the analysis without these participants (all *p* > .05; also see analysis of memory retention below).

### Memory retention

**Fig 2:**
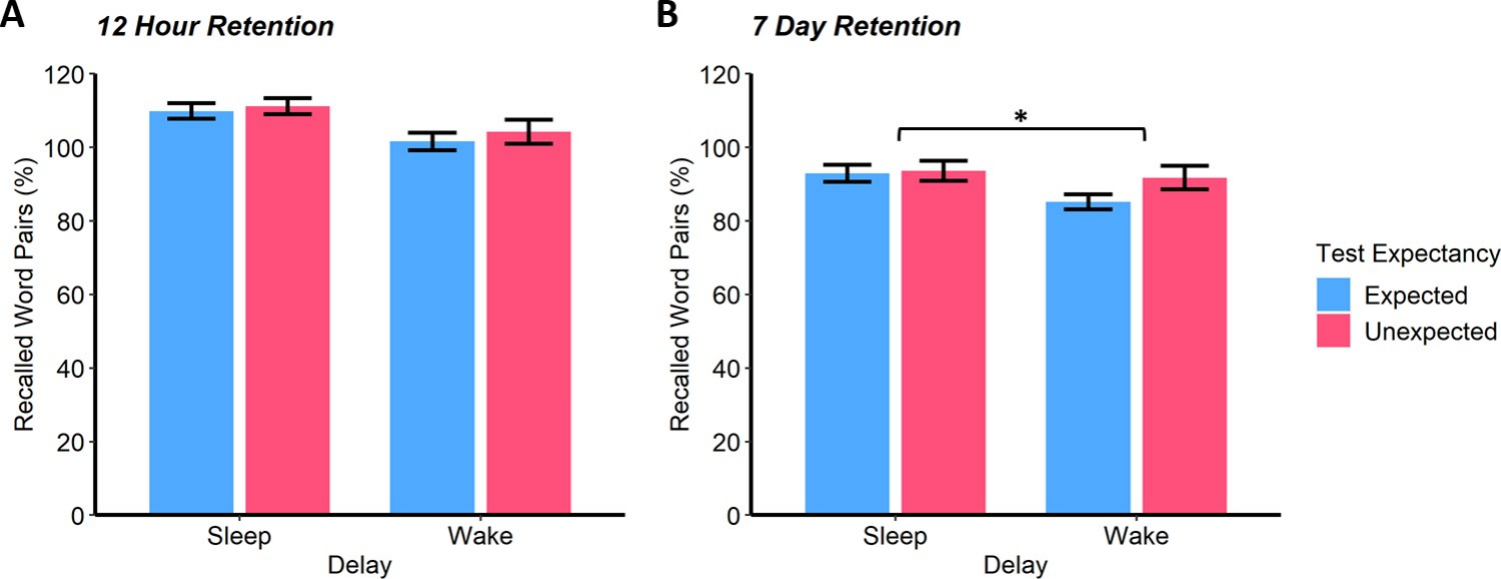
Experiment 1 Memory retention. Performance at the 12-hour (A) and 7-day (B) tests in Experiment 1. Performance is indicated by the percentage of word pairs recalled at each test, with performance on the final baseline test set to 100%. Data are shown as mean ± SEM; *p < .05, **p < .01.

Memory retention at the 12-hour test was better after sleep than wakefulness (*F*(1, 158) = 9.67, *p* = .002, *η*_*p*_^*2*^ = 0.06, Fig 2A). However, performance was not influenced by knowledge of a future test (*F*(1, 158) = 0.63, *p* = .428) and there was no Delay*Expectancy interaction (*F*(1, 158) = 0.07, *p* = .786). Sleep-associated memory benefits were not therefore amplified for individuals who expected a test. In line with this interpretation, our Bayesian ANOVA yielded a Delay*Expectancy *BF*_01_ of 4.00, indicating moderate evidence in favour of the null hypothesis.

The same pattern of results was observed at the 7-day test (Fig 2B), with an overall benefit of sleep on memory retention (*F*(1, 158) = 4.30, *p* = .040, *η*_*p*_^*2*^ = 0.03). Knowledge of a future test did not influence performance (*F*(1, 158) = 1.96, *p* = .163) and there was no Delay*Expectancy interaction (*F*(1, 158) = 1.34, *p* = .249, *BF*_01_ = 2.47).

These results remained the same when participants who did not meet the 60% baseline performance criterion were removed from the analysis. Only a main effect of Delay was observed at the 12-hour (*F*(1,149) = 16.93, *p* < .001, *η*_*p*_^*2*^ = 0.10) and 7-day test (*F*(1,149) = 4.68, *p* = .032, *η*_*p*_^*2*^ = 0.03) was observed. There were no main effects of Expectancy and no Delay*Expectancy interactions at either test (all *p* > .05).

To capture the change in retention between the 12-hour and 7-day tests, memory retention at the 7-day test was re-analysed, with performance in the 12-hour test set to 100%. When analysed with the same ANOVA as described above, there were no differences between conditions (Delay: *F*(1, 158) = 0.95, *p* = .330; Expectancy: *F*(1, 158) = 0.99, *p* = .321), and no Delay*Expectancy interaction was observed (F(1, 158) = 2.25, *p* = .136).

### Self-reported sleep

Participants in the sleep groups reported the number of hours that they had slept between encoding and the 12-hour test. Mean (± SEM) hours slept was comparable between the sleep-expected (6.58 ± 0.25) and the sleep-unexpected (7.01 ± 0.21) conditions (*t*(79) = 1.28, *p* = .203). To ensure that our results were not influenced by poor sleepers (i.e. those who reported less than 6 h of sleep) we repeated our main analyses without these participants (n = 13). The foregoing findings were replicated, with only a significant memory benefit of sleep emerging at the 12-hour (*F*(1, 145) = 10.15, *p* = .002, *η*_*p*_^*2*^ = 0.07) and 7-day test (*F*(1, 145) = 4.27, *p* = .041, *η*_*p*_^*2*^ = .03). All other effects and interactions were non-significant (*p* > .05).

No significant correlation was observed between self-reported sleep and task performance in either the sleep-expected (12-hour test: *r* = 0.06, *p* = .686; 7-day test: *r* = 0.05, *p* = .779) or sleep-unexpected condition (12-hour test: *r* = 0.20, *p* = .200; 7-day test: *r* = 0.26, *p* = .109).

### Alertness

Alertness levels, as indicated by the SSS (Table 1) were comparable across the Delay and Expectancy conditions at the baseline test and 7-day test (all *p* > .05). Interestingly, at the 12-hour test, the sleep group rated themselves as less alert than the wake group (*F*(1, 158) = 26.55, *p* < .001, *η*_*p*_^*2*^ = 0.14). This pattern of results is however contrary to what would be expected if alertness levels were driving the retention advantage for the sleep (vs wake) groups, and therefore cannot explain the purported benefit of sleep for memory. There was no main effect of Expectancy and no Delay*Expectancy interaction at the 12-hout test (all *p* > .05).

**Table 1:** Stanford Sleepiness Scale (SSS) scores for each condition and each session of Experiment 1.

	Baseline		12-hour test		7-day test	
	Expected	Unexpected	Expected	Unexpected	Expected	Unexpected
**Sleep**	2.64 ± 0.14	3.00 ± 0.11	3.24 ± 0.24	3.51 ± 0.23	3.02 ± 0.22	3.44 ± 0.25
**Wake**	2.94 ± 0.14	2.94 ± 0.25	2.24 ± 0.11	2.45 ± 0.22	2.90 ± 0.11	3.07 ± 0.20

Data are presented as means ± SEM.

Performance in the PVT (Table 2) was comparable across the Delay and Expectancy conditions at the baseline and 12-hour tests (all *p* > .05). At the 7-day test, we observed a Delay*Expectancy interaction (*F*(1,158) = 7.69, *p* = .006, *η*_*p*_^*2*^ = 0.05). Post-hoc comparisons show that this effect was driven by a greater number of attentional lapses in the wake-expected condition compared to the sleep-expected condition (*p*_*bonf*_ = .015). No other post-hoc comparisons were significant (*p*_*bonf*_ > .05). There were no main effects of Delay or Expectancy at the 7-day test (all *p* > .05).

**Table 2:** Percentage of PVT trials with attentional lapses (RT > 500 ms) in Experiment 1.

	Baseline		12-hour test		7-day test	
	Expected	Unexpected	Expected	Unexpected	Expected	Unexpected
**Sleep**	7.19 ± 1.32	9.81 ± 2.32	11.64 ± 2.05	11.57 ± 1.65	10.40 ± 2.26	14.82 ± 1.76
**Wake**	10.03 ± 2.08	8.86 ± 1.97	13.24 ± 2.63	9.52 ± 2.30	20.38 ± 2.90	11.04 ± 2.14

Data are presented as means ± SEM.

## Experiment 2

In Experiment 1, we failed to replicate the finding that sleep selectively strengthens memories that are deemed relevant for the future [24]. One potential explanation for this failed replication is that baseline performance levels were too high to allow knowledge of a future test to augment sleep-associated consolidation. Baseline memory performance in our first experiment was 77%, although true performance levels were likely far higher due to the feedback provided at the final baseline testing round. Indeed, accuracy levels at the 12-hour test exceeded baseline performance despite there being no further exposure to the word pairs. To address the possibility that memories were encoded too strongly to permit a selective benefit of sleep on future-relevant information, we ran a second, pre-registered experiment (osf.io/3k6ej) in which baseline performance levels were reduced relative to Experiment 1.

### Methods

#### Participants

Two-hundred and thirteen adults (126 women, mean ± SD age: 24.45 ± 3.86 years) were recruited using the same procedures as described in Experiment 1. As before, only participants who expected/did not expect a memory test in line with their respective conditions were included in our analysis (a total of 82 participants were excluded on this basis, sleep-expected *n* = 5, sleep-unexpected *n* = 32, wake-expected *n* = 8, wake-unexpected *n* = 37). A further seven participants were excluded from the analysis for either taking a daytime nap during the ‘wake’ delay of the experiment (*n* = 6) or reporting to have obtained no sleep during the ‘sleep’ delay (*n* = 1). This resulted in a final sample of 124 participants (78 women, mean ± SD age: 24.32 ± 4.05 years) who were allocated equally across conditions (*n* = 31 in each condition).

#### Procedure

There were three key modifications to the procedures: 1) the number of stimuli was increased from 40 to 100 word pairs, 2) the criterion participants were required to meet during the baseline assessment was reduced from 60% to 40% correct responses, and 3) only a 12-hour follow-up test was included (because no changes were observed between the 12-hour and 7-day tests in Experiment 1). Sixty-eight percent of participants met the baseline performance criterion on the first round of testing, whereas the remaining participants met this criterion on the second testing round. The proportion of participants who completed a second test did not differ between the sleep and wake groups, *X*^*2*^ (1, *N* = 124) = 1.83, *p* = .176.

## Results

### Baseline memory performance

A 2 (Delay: Sleep/Wake) * 2 (Expectancy: Expected/Unexpected) * 2 (Experiment: 1/2) ANOVA confirmed that baseline memory performance was significantly lower in Experiment 2 (64% ± 1.29; ± SEM) relative to Experiment 1 (77%; *F*(1, 278) = 65.08, *p* < .001, *η*_*p*_^*2*^ = 0.19, Table 3). All other main effects and interactions were not significant (*p* > .05), and this was also the case when baseline performance in Experiment 2 was assessed in isolation (Delay: *F*(1, 120) = 0.01, *p* = .917; Expectancy: *F*(1, 120) = 0.54, *p* = .464); Delay*Expectancy: (*F*(1, 120) = 0.005, *p* = .946).

**Table 3:** Baseline memory performance for each condition in Experiment 1 and Experiment 2. Performance was calculated as the percentage of correctly recalled word pairs at the final baseline testing round.

	Experiment 1		Experiment 2	
	Expected	Unexpected	Expected	Unexpected
**Sleep**	77.56 ± 1.58	77.12 ± 1.96	62.74 ± 2.37	64.84 ± 2.64
**Wake**	75.85 ± 1.94	75.92 ± 2.14	62.65 ± 2.65	64.39 ± 2.76

Data are presented as means ± SEM.

### Memory retention

**Fig 3:**
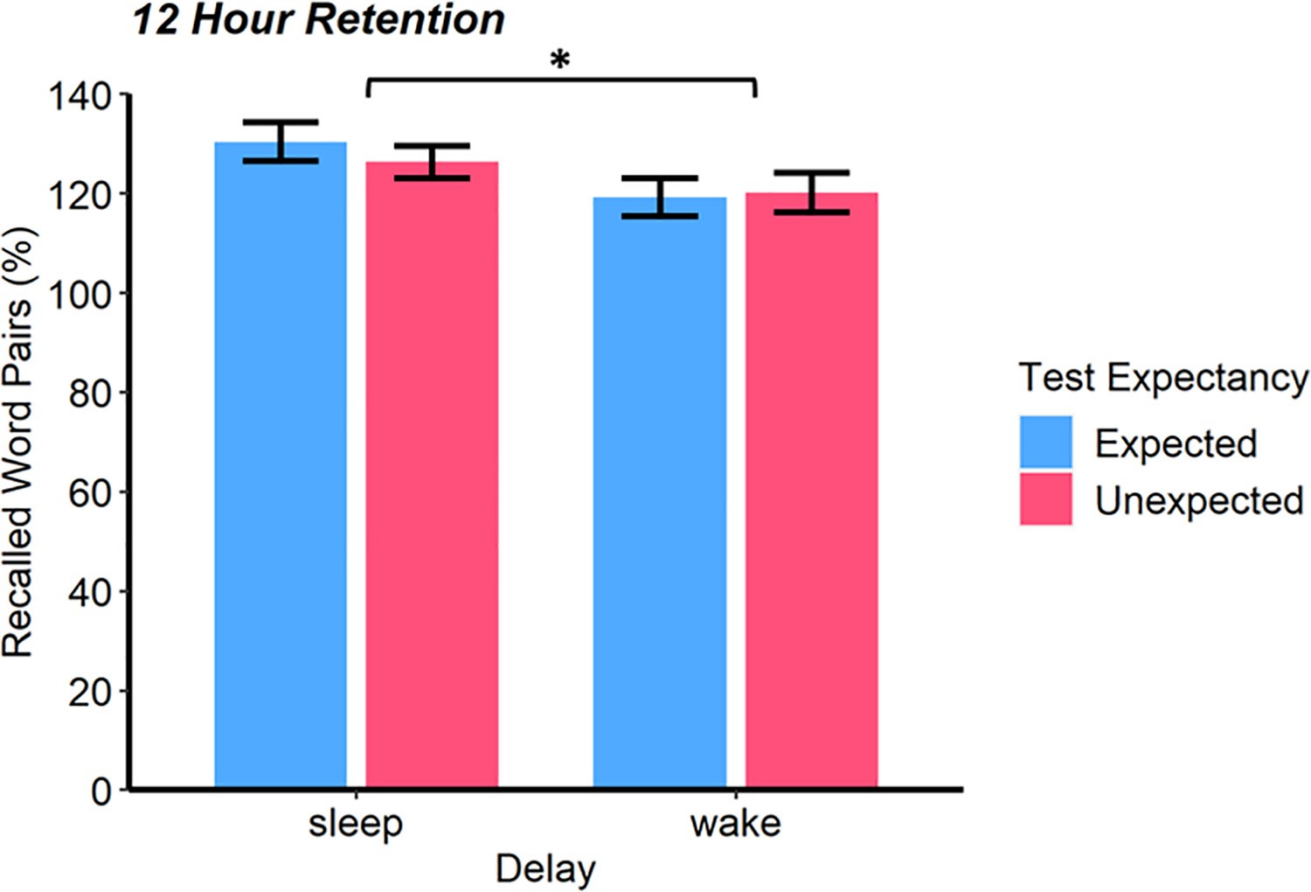
Experiment 2 Memory retention. Perfomance at the 12-hour test in Experiment 2. Performance is indicated by the percentage of word pairs recalled at test, with performance on the final baseline test set to 100%. Data are shown as means ± SEM; *p < .05.

Our findings mirrored those of Experiment 1. Memory retention at the 12-hour test was higher after sleep than wakefulness (*F*(1, 120) = 5.27, *p* = .023, *η*_*p*_^*2*^ = 0.04, Fig 3). However, performance was not influenced by knowledge of a future test (*F*(1, 120) = 0.17, *p* = .682) and there was no Delay*Expectancy interaction (*F*(1, 120) = 0.45, *p* = .504). A Bayesian ANOVA yielded a Delay*Expectancy *BF*_01_ of 3.42, indicating moderate evidence in favour of the null hypothesis. As before, our findings suggest that sleep-associated memory benefits were not amplified for individuals who expected a test.

### Self-reported sleep

Mean (± SEM) hours slept was comparable between the sleep-expected (7.05 ± 0.25) and the sleep-unexpected (6.69 ± 0.29) conditions (*t*(60) = 0.93, *p* = .359). Again, to ensure that our results were not influenced by participants who reported less than 6 h of sleep, we repeated our analyses without these participants (*n* = 9). Our findings were replicated, with only a significant memory benefit of sleep emerging from the ANOVA (*F*(1, 111) = 6.39, *p* = .013, *η*_*p*_^*2*^ = 0.05). There were no other significant main effects or interactions (all *p* > .05).

As before, there was no significant correlation between self-reported sleep and task performance in either the sleep-expected (*r* = 0.09, *p* = .633) or sleep unexpected condition (*r* = 0.11, *p* = .558).

### Alertness

SSS scores (Table 4) were comparable across conditions at the baseline test (all *p* > .05), however as observed in Experiment 1, at the 12-hour test participants in the sleep groups rated themselves as less alert than those in the wake groups (*F*(1,120) = 5.12, *p* = .026, *η*_*p*_^*2*^ = 0.04). There was no main effect of Expectancy and no Delay*Expectancy interaction in this analysis (all *p* > .05).

**Table 4:** Stanford Sleepiness Scale (SSS) scores for each condition and each session of Experiment 2.

	Encoding		12-hour test	
	Expected	Unexpected	Expected	Unexpected
**Sleep**	2.58 ± 0.13	2.58 ± 0.12	3.48 ± 0.29	3.26 ± 0.23
**Wake**	2.77 ± 0.19	2.84 ± 0.14	2.84 ± 0.21	2.81 ± 0.23

Data are presented as means ± SEM.

The number of attentional lapses made by participants in the PVT (Table 5) were equivalent across Delay and Expectancy conditions at both the baseline test and the 12-hour test, no main effects or interaction effects were observed at either session (all *p* > .05).

**Table 5:** Percentage of PVT trials with attentional lapses (RT > 500ms) in Experiment 2.

	Encoding		12-hour test	
	Expected	Unexpected	Expected	Unexpected
**Sleep**	9.81 ± 2.22	8.00 ± 2.08	22.26 ± 3.66	14.51 ± 3.09
**Wake**	11.36 ± 2.41	10.26 ± 2.06	15.55 ± 3.53	11.72 ± 2.30

Data are presented as means ± SEM.

## Discussion

In the current study, we sought to replicate the seminal finding that sleep preferentially strengthens memories that are deemed to be relevant for the future [24]. In Experiment 1, we ran an online replication of Wilhelm et al. [24], using a verbal paired associates paradigm. We observed a benefit of sleep for the retention of word pairs, but, unlike the original study, we found no evidence that sleep-associated memory effects were influenced by knowledge of a future test. In Experiment 2, we reduced pre-sleep memory performance to account for the potential impact of memory strength on overnight consolidation [17,36,37,44–46]. We replicated the results of Experiment 1, observing an overall memory benefit of sleep, but not a selective influence of sleep on future-relevant information.

To our knowledge, this is the first study to attempt a direct replication of Wilhelm et al. [24] using the same paired associates protocol. Although our results do not support the original findings, they are in keeping with a growing number of studies that have also failed to observe an amplified benefit of sleep on future-relevant information [34,35]. The finding that sleep *does not* offer a selective memory benefit for future relevant information has therefore been shown across a number of different declarative and non-declarative memory paradigms. These findings also complement a broader literature, where selective sleep-associated memory effects are not always replicated. For example, emotionally salient information is widely considered to be particularly sensitive to consolidation during sleep [47], but many studies have failed to find a preferential impact of sleep on emotionally negative relative to neutral memories [6,26–29,48]. To what extent, and under what conditions, sleep offers selective benefits to memory is therefore still largely unknown.

Previous studies suggest that the memory benefits of sleep are amplified for weakly-encoded relative to strongly-encoded information [17,36,37,45,46,49,50]. We therefore reasoned that baseline performance levels in Experiment 1 may have been too high to allow knowledge of a future test to augment sleep-associated consolidation. To address this issue, we modified our task in a second experiment to reduce performance at baseline. Mirroring Experiment 1, memory retention was superior after sleep than wakefulness, but this effect was comparable for individuals who did and did not expect a future test. Hence, memory strength at encoding does not appear to explain our failure to replicate the findings of Wilhelm et al. [24].

It is nevertheless important to emphasise that our study was carried out in an online environment and not in a laboratory. The experimental control afforded by a laboratory may have had a major impact on how sleep influenced memory consolidation in the original investigation by Wilhelm et al. [24]. Of particular importance was the physical presence of an experimenter, which would have presumably increased the salience of instructions pertaining to future tests. Other factors that can be more readily measured in the laboratory include the time between encoding and bed time, overall sleep time and sleep architecture. Although online questionnaires can provide some insight into participant sleep practices, they do not offer the same degree of precision as objective laboratory measures such as polysomnography. Our findings therefore demonstrate that a selective overnight strengthening of future-relevant information does not emerge in an online environment, although further work is necessary to determine whether such effects replicate in the laboratory.

Beyond the experimental environment, there were also several minor procedural differences between this study and Wilhelm et al. [24], which might have influenced our findings. Notably, we did not attempt to control the time between completion of the experimental tasks and sleep onset. There is evidence to suggest that sleep-memory effects are amplified when sleep closely follows learning [17,51,52], and a shorter delay between learning and sleep might also facilitate the selective strengthening of future-relevant information. Further procedural differences between our study and Wilhelm et al. include our use of an explicit encoding instruction (i.e. we asked participants to imagine the words of each pair interacting whereas Wilhelm et al did not report doing so), and our puzzle matching (interference) task, which was shorter in duration than the video game used by Wilhelm et al. It is possible that these procedural differences reduced the sensitivity of our protocol to selective memory strengthening in sleep.

Our results are however, in keeping with an emerging body of online research investigating sleep-associated consolidation. For example, Kroneisen & Kuepper-Tetzel [51] also observed a benefit of overnight sleep (vs daytime wakefulness) on the retention of verbal paired associates using a web-based task. To our knowledge, we are the first to show in an online study that the memory effects of sleep are retained across a longer retention interval of 7 days. This effect is in line with numerous laboratory studies that have shown long-term benefits of sleep for memory [53–55] and supports the view that sleep promotes the retention of durable and long-lasting memory representations [1–3].

A general concern in studies comparing memory retention after overnight sleep and daytime wakefulness is that participants may be more alert in the morning after sleep, as compared to the evening after wakefulness, meaning that an apparent memory benefit of sleep could be driven by between-condition differences in alertness. However, in both experiments of the current study, the sleep groups rated themselves as *less alert* than the wake groups when they returned to complete the test phase. This is precisely the opposite pattern of results that would be expected if between-group differences in alertness could explain the purported memory effect of sleep. We cannot rule out an impact of other circadian factors pertaining to evening and morning testing, particularly given that participants were allocated to their respective conditions based on the time of day that they signed up for the study (although they were unaware of the crucial sleep vs wake manipulation). Many other studies have however reported superior retention after a daytime nap relative to a corresponding interval of wakefulness, suggesting that sleep-associated memory benefits are not driven by time-of-day effects.

In sum, we were unable to replicate the seminal finding that sleep selectively strengthens memories that are deemed relevant for the future [24]. Our findings, derived from two online experiments, are in keeping with a number of recent studies that have also failed to observe a targeted effect of sleep on future-relevant information [34,35]. However, given the important differences in experimental control between our online study and the original laboratory experiment of Wilhelm et al. [24], further work is necessary to determine the conditions under which these selective sleep-memory effects may emerge. Nevertheless, our findings add to a developing body of online research indicating that sleep provides robust and long-lasting benefits for memory [56].
